# Development and validation of a food frequency questionnaire (FFQ) for assessing dietary macronutrients and calcium intake in Cambodian school-aged children

**DOI:** 10.1186/s12937-019-0437-3

**Published:** 2019-02-21

**Authors:** Yoko Horiuchi, Kaoru Kusama, Kanha Sar, Nobuo Yoshiike

**Affiliations:** 1 Graduate School of Health Sciences, Aomori University of Health and Welfare, 58-1 Mase, Hamadate, Aomori City, Aomori, 030-8505 Japan; 2 Department of Food and Health Sciences, Faculty of Health and Human Development, The University of Nagano, 8-49-7 Miwa, Nagano City, Nagano, 380-8525 Japan; 3 Strengthening Public Health Laboratory, Workforce Development, and Health Systems Research Capacity of the Cambodian National Institute of Public Health, Lot #80, St. Samdach Penn Nouth Blvd (289), Boeung Kok 2 commnue, Toul Kork district, Phnom Penh, 12152 Cambodia

**Keywords:** Food frequency questionnaire, School-aged children, Cambodia, Validity, Development

## Abstract

**Background:**

The nutritional status of school-aged children in Cambodia remains largely unknown. No tools for large-scale assessment of daily nutrient intake exist for this population, making development of appropriate intervention strategies difficult. Thus, we aimed to devise and validate a food frequency questionnaire (FFQ) that is suitable for and dedicated to assessing the dietary intake of macronutrients and calcium in school-aged children in Cambodia.

**Methods:**

We developed an FFQ based on data from a single 24-h recall survey of 2020 children. The final list, which was developed as specified in the Block method and stepwise multiple regression analysis, comprised of 56 food items covering intake of energy, macronutrients, and calcium. We assessed the validity of the FFQ by comparison with a duplicated 24-h recall survey before and after de-attenuation. We also tested the reproducibility by comparing the first and second FFQs (FFQ1 and FFQ2) administered at an interval of approximately 6 weeks.

**Results:**

The 56 food items in the FFQ accounted for 73.3% of the dietary calcium intake of Cambodian children and explained most of the inter-individual variation (cumulative R^2^: 0.96). The intake estimated by the FFQ was lower than the average intake across the two 24-h recall surveys. Spearman’s correlation coefficients for comparison between FFQ1 and FFQ2 ranged from 0.29 for fat to 0.47 for calcium. After de-attenuation of data, Pearson’s correlation coefficients ranged from 0.38 for fat to 0.71 for energy. Cross-classification analysis indicated that the average percentage of the subjects classified in the same or adjacent quartiles was 78.0%.

**Conclusions:**

The FFQ is potentially a reliable scale for measuring nutrient intake in this population.

## Background

The nutritional status and diet of school-aged children in Cambodia remain unknown due to the lack of large-scale nationwide surveys, because children under 5 years old in this country had been given priority. To rectify this lack of information, the Foundation for International Development/Relief (FIDR) started a project that included a nationwide nutritional survey of the country’s children in collaboration with the Department of Preventive Medicine of the Cambodian Ministry of Health between November 2014 and July 2015, with a sample that included 2020 children aged 6–17 years old. The results revealed that the children’s body weight was lower than the reference values, and the children’s intake of energy, macronutrients, and other selected nutrients did not meet the Recommended Dietary Allowances in Cambodia (CAM-RDA) in all subgroups for age and sex [1]. In particular, the percentage of children consuming calcium below the CAM-RDA were 86.1% [1].Therefore, nutritional interventions focusing on increased calcium intake, in addition to the energy and macronutrients specific to Cambodian school-aged children, need to be initiated through appropriate nutrition-education programs [1]. However, there are no tools for the measurement of the daily nutrient intake (including calcium) of Cambodian school-aged children that can be widely adopted for large target populations to evaluate the effectiveness of such interventions. Optimal calcium intake by children and young adults is related to achieving peak adult bone mass [2]. Because of the important role of calcium in childhood health, a reliable and feasible tool for assessing calcium intake is needed [3].

There are several methods for estimating dietary calcium intake, including the 24-h recall method (24H), dietary history record (DR), and food weight record (FR). Although these methods are generally accepted as providing valid information about a person’s dietary intake [4], the DR and FR are quite burdensome, further, both the 24H and the FR are unsuitable for large-scale epidemiological studies because of the time and level of literacy required, as well as economic constraints on data collection [5]. On the other hand, the food frequency questionnaire (FFQ) has been shown to be the most useful method of assessing food or nutrient intake in epidemiological studies, because it is quicker to complete and involves less work for both interviewers and participants, as well as being relatively inexpensive [5]. During the last 15 years, various FFQs have been developed and validated to assess dietary intake of calcium and other nutrients in countries around the world [3, 6–21]. It has been reported that there is a wide variation of calcium intake between nations, depending largely on the consumption of dairy products [22]. Therefore, we developed and validated a new FFQ to assess dietary intake of calcium and macronutrients by Cambodian school children.

## Methods

### Development of the FFQ

We designed an FFQ to assess Cambodian children’s dietary intake of calcium and macronutrients to monitor growth and nutritional conditions in the target populations. The questionnaire was developed based on the dietary data using the 24-h recall method in the survey for school-aged children in Cambodia. A total of 2048 randomly sampled children (6–17 years old) were recruited and valid data for 2020 of these subjects (959 boys and 1061 girls) were collected [1]. Nutrient analysis was performed using the FIDR Nutrition Calculation Database 2013, which was developed based on the Association of Southeast Asian Nations (ASEAN) Food Composition Tables [23] and Sustainable Micronutrient Interventions to Control Deficiencies and Improve Nutritional Status and General Health in Asia project (SMILING) food composition table for Cambodia [24]. Through the following three steps, we selected food items for the FFQ from among a total of 595 foods that were listed in the dietary data obtained from 2020 subjects. First, the recalled foods were ranked according to the Block method [25]. Then, we selected 101 foods that make up 80% of the total population’s nutrient intake, excluding duplicate foods. There were 42 food items contributing up to 80% of total energy intake, as well as 55, 44, 20, and 56 food items contributing up to 80% of protein, fat, carbohydrate, and calcium intake, respectively. Second, using stepwise multiple regression analyses, 78 food items in the model that make up 0.99 of calcium intake of the between-person variability were considered eligible for the FFQ. We calculated the cumulative percent contribution to calcium intake and the cumulative R^2^ for calcium, as shown for the top 15 items in Table 1. Third, before and after the regression analysis, we classified all food items into 15 large, 31 medium, and 85 small groups based on their nutritional content; food items with similar nutritional content were grouped together as one food item. Taking into account the dietary habits of children in Cambodia, we also added five items (green banana, pork blood, oil, salt, and snacks). In addition, cheese and yogurt were added because of their calcium content, although they do not make a significant contribution to the dietary calcium intake in this population. Finally, a total of 56 food items were determined, with 14 food groups (Table 2). This number of items is similar to other FFQ’s designed for schoolchildren [11]. The cumulative percent contribution to calcium intake and cumulative R^2^ for calcium in these 56 food items were 73.3% and 0.96, respectively. Frequency of intake was evaluated based on the intake over the previous one month. For each item, 10 response categories were provided with a choice of frequencies: never, once a month, 2–3 times a month, 1–2 times a week, 3–4 times a week, 5–6 times a week, once a day, 2–3 times day, 4–5 times a day, or 6 times or more a day. For boiled rice only, the choice of frequency was set as follows: once a day, 2–3 times a day, 4–5 times a day, or 6 times or more a day. The portion for each item was calculated by using the median intake determined in the based survey and took into account the typical values and natural units of foods from the market. Finally, portion size options were given using standard measuring units (e.g. bag, cup, dish, spoon, slice, bowl, or piece) by referring to photographs provided that were representing sizes.

**Table 1:** Cumulative contribution and cumulative R^2^ for calcium intake in the FFQ (top 15 of 56 items)

Ranking	Food item	Cumulative contribution %	Ranking	Food item	Cumulative R^2^
1	Fish paste	19.4	1	Fish paste	0.37
2	Boiled rice	30.2	2	Fermented fish	0.50
3	Fish sauce	34.0	3	Snails	0.60
4	Fermented fish	37.7	4	Condensed milk	0.65
5	Snakehead fish	41.3	5	Crab	0.69
6	Condensed milk	44.2	6	Fish sauce	0.73
7	Duck egg	47.0	7	Fresh milk	0.76
8	Soft drink juice	49.7	8	Ice cream	0.79
9	Fresh milk	51.6	9	Soft drink juice	0.81
10	Morning glory	53.4	10	Dried shrimp	0.83
11	Ice cream	55.0	11	Boiled rice	0.84
12	Soft drink, tea	56.6	12	Sweet potato	0.86
13	Bread	58.0	13	Shrimp	0.87
14	Snails	59.4	14	Bread	0.88
15	Soybean milk	60.6	15	Soybean milk	0.89

*FFQ* food frequency questionnaire

**Table 2:** The 56-food list of the developed FFQ to assess the dietary intake of energy, macronutrients, and calcium of school-aged children in Cambodia

Food group	Food name
1-Cereal and cereal product	Boiled rice, Rice noodle, Bread, Wheat noodle, Corn
2-Tuber and starchy	Sweet potato
3-Vegetables	Morning glory, Amaranth, Spinach, Cucumber, Bean sprout
4-Fruits	Yellow banana, Green banana, Coconut cream, Guava, Green mango, Ripe papaya, Spanish plum, Rambutan,
5-Legume and nuts	Soybean fermented, Soybean milk, Boiled mung bean, Roasted peanut
6-Meats and meat products	Beef, Pork, Beef meat ball, Chicken, Pork blood,
7-Egg	Duck egg
8-Fish and Selfish	Snakehead fish, Clam, Snail, Shrimp, Crab, Canned fish, Dried shrimp
9-Milk and milk product	Fresh milk, Yogurt, Yogurt drink, Cheese, Milk powder, Ice cream, Condensed milk
10-Confectionary	Rice cake, Snack, Mung bean cake
11-Beverage	Milo powder, Ovaltine powder, Soft drink juice, Soft drink tea,
12-Fats and oils	Oil
13-Sugars and syrup	Sugars
14-Condiments and spice	Fish paste, Fish sauce, Salt, Fermented fish

*FFQ* food frequency questionnaire

### Validation study

#### Study participants

A total of 121 students (53 boys and 68 girls) aged 6–17 years were recruited based on the two-stage systematic cluster sampling. In the first stage, each school in 4 communities of Sen sok, tuol Kauk, Meanche, and Chamkar Morn was selected via a systematic random sampling technique from seven districts of the Department of Education in Phnom Penh city. In the second stage, from each school, sampling numbers of students was performed using the proportional cluster sampling method.

### Data collection

#### Anthropometric assessment

Anthropometric measurements of the child were obtained using standardized techniques and calibrated equipment. Height was measured to the nearest 0.1 cm with the child bare footed, using a stadiometer. Body Mass Index was calculated as weight (kg)/height (m^2^). All measurements were carried in duplicates and the average was used in the analysis.

#### Dietary intake assessment

For the 24-h recall method, a quick dietary record form was used to record the foods and drinks that respondents consumed during the day. The form for the FFQ was used to assess the dietary intake of respondents. A photograph that showed the standard portion size of each food item was employed to help respondents easily point out different portion sizes. The FFQ was repeated about 6 weeks later in order to evaluate its reproducibility (the first and the second FFQ are denoted hereafter as FFQ1 and FFQ2). Along with this reproducibility study, the FFQ was validated by referring to the 24H at the same time interval. FFQ1 was performed in December 2016 and FFQ2 was performed in January 2017. At both rounds, after the 24H was conducted, the FFQ was completed with each respondent by the trained interviewer. We also measured each respondent’s height and weight before each round. Fourteen students were absent for the second round and therefore valid data were collected for only 107 respondents (48 boys and 59 girls).

### Statistical analysis

Anthropometric indices were calculated using the World Health Organization (WHO) 2006 standards (ANTHRO version 3.2.2 January 2011, Geneva, Switzerland) and expressed as height-for-age (HAZ) and body mass index for age (BAZ).

Descriptive results were expressed as means and standard deviations, or as percentage of and frequencies for continuous and qualitative variables, respectively for all nutrients from the duplicated FFQ and the 24H. Reproducibility was assessed by calculating correlations between these intakes derived from FFQ1 and FFQ2. Validity was assessed by comparison with FFQ1 and with the average of the two 24H surveys (24Hs). Nutrient values were subjected to log-transformation, if necessary. Spearman’s correlation coefficients were calculated using the crude nutrient data. Pearson’s correlation coefficient was calculated after statistical adjustments (energy-adjusted and de-attenuation). Energy-adjusted nutrient intakes were calculated as residuals from the regression with nutrient intakes as dependent variables and with energy as an independent variable. De-attenuated correlations were also calculated to remove within-person variance (i.e., day-to-day variation) from the 24H by using the following formula: (1+ (S^2^w/S^2^b) n)^0.5^ where n represents the number of replicate measurements (*n* = 2), S^2^w is the within-person variation, and S^2^b is the between-person variation [5]. Additionally, cross-classification analysis was performed to determine whether there was good agreement, and to estimate the percentage of subjects classified into the same or an adjacent quartile for each nutrient. All analyses were carried out using the Statistical Package for the Social Sciences version 24 (SPSS Inc., Chicago, IL, USA).

## Results

Table 3 shows the characteristics of study participants at the time of the second FFQ. Participants had a mean age of 11.24 years (± 2.75 years) and 44.9% were boys. The percentage of participants with z-score of − 2 standard deviations in z-score was 9.4% for height for age and 5.6% for body mass index (BMI) for age.

**Table 3:** Characteristics of study participants completing the second food frequency questionnaire among the school-aged children in Cambodia (*n* = 107)

Sex	Boys (*n* = 48)	Girls (*n* = 59)	Total (*n* = 107)
Age (years)^a^	10.90 ± 2.58	11.53 ± 2.88	11.24 ± 2.75
HAZ z-score^a^	−0.69 ± 1.06	−1.08 ± 1.06	−0.91 ± 1.07
BAZ z-score^a^	−0.31 ± 1.40	−0.49 ± 1.04	−0.41 ± 1.21
HAZ z-score < −2SD, n (%)^b^	2 (4.12)	8 (13.56)	10 (9.35)
BAZ z-score < −2SD, n (%)^b^	3 (6.23)	3 (5.08)	6 (5.61)

^a^Data are mean ± standard deviation. *SD* standard deviation

*BMI* body mass index, weight (kg)/height (m^2^), *HAZ* Height for age, *BAZ* BMI for age

^b^n number (percentage to all boys *n* = 48, all girls *n* = 59, total *n* = 107)

Table 4 shows the mean nutrient intakes and standard deviations derived from the FFQ1, FFQ2, and 24Hs, as well as the mean percent differences between these assessments. The FFQs tended to underestimate the intake of all nutrients compared to the 24Hs. The mean difference between the two FFQs ranged from 0% for fat to − 6% for energy and protein. For de-attenuation, we calculated the ratio of the components of within-person and between-person variability, which ranged from 1.3 for calcium to 2.1 for energy.

**Table 4:** Comparison of energy and nutrient intakes obtained from the two FFQs and 24Hs among the school-aged children in Cambodia (*n* = 107)

Energy and nutrients	Mean (standard deviation)			% mean difference		Variance ratio^c^
	FFQ1	FFQ2	24Hs	FFQ1 vs. FFQ2^a^	FFQ1 vs. 24Hs^b^	S^2^w/S^2^b^d^
Energy (kcal)	1215 (472)	1073 (368)	1783 (492)	− 6	−30	2.1
Protein (g)	33.6 (17.5)	28.0 (12.0)	54.6 (16.8)	−6	−37	1.9
Fat (g)	19.3 (12.7)	16.2 (10.3)	36.3 (14.7)	0	− 39	1.5
Carbohydrates (g)	225.5 (83.7)	203.1 (69.0)	309.0 (87.7)	−5	−25	1.7
Calcium (mg)	345 (254)	315 (478)	450 (242)	1	−17	1.3

^a^ % mean difference for FFQ1 vs. FFQ2 individually calculated as: (FFQ2-FFQ1)/FFQ1 × 100

^b^ % mean difference for FFQ1 vs. 24Hs individually calculated as: (FFQ1-24Hs)/24Hs × 100

^c^Variance ratios were calculated from the estimated values of two 24-h recall surveys conducted in 107 children from Phnom Penh, Cambodia

^d^S^2^w: within-person variation, S^2^b: between-person variation

*FFQ* food frequency questionnaire, *FFQ1* the first FFQ, *FFQ2* the second FFQ, *24Hs* the mean of the two 24-h recall surveys (24Hs)

Table 5 shows the correlations between FFQ1 and FFQ2. For all subjects, Spearman’s correlation coefficients for crude daily nutrients ranged from 0.29 for fat to 0.47 for calcium, while the coefficients for energy-adjusted nutrients ranged from 0.23 for protein to 0.41 for calcium. In addition, the correlation coefficients for both crude and energy-adjusted values were higher in boys than in girls, as well as in children aged 10–17 years than in those aged 7–9 years (data not shown).

**Table 5:** FFQ reproducibility: Spearman’s correlation coefficient analysis of agreement and cross-classification analysis for energy and nutrient intakes as reported in FFQ1 and FFQ2 among the school-aged children in Cambodia (*n* = 107)

Energy and nutrients	Spearman's correlation coefficients		% classified into same ±1 quartile^b^	% Misclassified^b^
	Crude	Energy-adjusted^a^		
Energy	0.44^**^	–	77	6
Protein	0.40^**^	0.23^*^	78	5
Fat	0.29^**^	0.32^**^	73	6
Carbohydrates	0.46^**^	0.29^**^	81	3
Calcium	0.47^**^	0.41^**^	82	3

*Significant (*p* < 0.05), **Highly significant (*p* < 0.01)

^a^Adjustment for energy was done by the residual method

^b^Cross-classification analyses were completed to validate agreement between FFQ1 and FFQ2 in terms of the proportions of participants classified into the same ±1 quantile or misclassified. *FFQ* food frequency questionnaire, *FFQ1* the first FFQ, *FFQ2* the second FFQ

The proportion of individuals correctly classified into the same or an adjacent quartile was the highest for calcium (82%) and lowest for fat (73%). Only a small percentage of the subjects were grossly misclassified (3–6%).

Table 6 compares the nutrient intake data obtained with the FFQ1 and 24Hs. Pearson’s correlation coefficients for the transformed values ranged from 0.29 for fat to 0.51 for carbohydrates. The de-attenuated and transformed values ranged from 0.38 for fat to 0.71 for energy. Furthermore, the correlation coefficients for the transformed and de-attenuated values, except those of calcium, were higher in boys than in girls, as well as in children aged 7–9 years than in those aged 10–17 years (data not shown).

**Table 6:** Validity of FFQ: Pearson’s correlation coefficient analysis of agreement and cross-classification analysis for energy and nutrient intakes as reported in FFQ1 and 24Hs among the school-aged children in Cambodia (n = 107)

Energy and nutrients	Pearson's correlation coefficient				% classified into same ±1 quartile^d^	% Mis-classified^d^
	Crude	Energy-adjusted^a^	Transformed^b^	De-attenuated transformed^c^		
Energy	0.53^**^	–	0.50^**^	0.71	81	7
Protein	0.50^**^	0.33^**^	0.44^**^	0.62	79	6
Fat	0.33^**^	0.18	0.29^**^	0.38	68	6
Carbohydrates	0.53^**^	0.31^**^	0.51^**^	0.70	81	5
Calcium	0.50^**^	0.46^**^	0.45^**^	0.59	81	6

*Significant (*p* < 0.05), **Highly significant (*p* < 0.01)

^a^Adjustment for energy was done by the residual method

^b^Transformed: values were log-transformed

^c^De-attenuated: values calculated as the ratio of within-person to between-person variation estimated in the two 24-h recall surveys. The formula for calculating de-attenuated values was (1+ (S^2^w/S^2^b) n) ^0.5^, where S^2^w is within-person variation and S^2^b is between-person variation. *n* = 2

^d^Cross-classification analyses were completed to validate agreement between FFQ1 and 24Hs in terms of the proportions of participants classified into the same ±1 quantile or misclassified. *FFQ* food frequency questionnaire, *FFQ1* the first FFQ, *24Hs* the mean of the two 24-h recall surveys

For all subjects, the cross-classification analysis demonstrated that the proportion of subjects correctly classified into the same or an adjacent quartile was the highest for energy, carbohydrates, and calcium (81%), while it was the lowest for fat (68%). Only a small percentage of the subjects were grossly misclassified (5–7%).

## Discussion

### Development of an FFQ for school-aged children

We developed an FFQ comprising 56 food items and determined that fish paste accounts for the highest percentage of dietary calcium intake (19.4%), followed by boiled rice (10.8%), as shown in Table 1. These findings reflect the dietary habits of Cambodians and are similar to FFQ data obtained in Vietnam, where rice was the second-highest contributor to calcium intake [21]. The contribution of dairy foods to calcium intake was low, but dairy foods had a high cumulative R^2^ for inter-individual variation of calcium intake. In the future, nutritional education programs in Cambodia should recommend higher intake of foods with a high calcium content.

### Study design

The validity and reproducibility of our FFQ were examined using the 24H as a reference point. In theory, the measurement errors of FFQ and the reference method should be independent: 64% of 227 validation studies used only one reference method, and 22% used a repeated 24H as a reference point [26].

### Reproducibility and validity of the FFQ

The absolute nutrient values obtained by the FFQ tended to be lower than those obtained by the 24H. This finding differed from the results of studies performed in other countries [3, 10–20], although calcium intake was lower according to the FFQ in some studies [8, 9]. These results may have been affected by the smaller numbers of food items in those FFQs, which were 10 [8] and 30 [9]. With respect to fat intake, because our survey inquired about respondents’ consumption of stir-fried dishes or fried dishes for oil intake, it may have been difficult for children to answer.

The correlations between FFQ1 and FFQ2 were moderate for all nutrients, except fat.

Spearman’s correlation coefficient for calcium (energy-adjusted value) was lower than in previous studies [14, 16]. The amounts of nutrients from FFQ1 were greater than those from FFQ2. This finding correlates with findings from other studies, which have reported higher nutrient intakes from FFQ1 compared to FFQ2 [10, 13–16].

The reproducibility was higher in children aged 10–17 years than in those aged 7–9 years. This result was consistent to that of a previous study [14] that showed the tendency of a higher correlation among high school students than among elementary school students. This could be because the knowledge about foods or dishes was greater among older children [27].

With respect to validation of FFQ1 by using the 24Hs, there was a strong correlation for energy and carbohydrate intake and a relatively good correlation for calcium intake by Pearson’s correlation coefficient analysis of crude and transformed daily nutrients (a correlation coefficient ≥ 0.50 is defined as satisfactory or good, 0.30–0.49 as fair, and < 0.30 as poor) [15, 28]. In addition, Pearson’s correlation coefficients between FFQ1 and the 24Hs for transformed values ranged from fair to satisfactory, except for fat. The observed moderate agreement could be due to errors in portion size estimation, and limitations of recall could also have contributed to the moderate agreement between the two methods [16].

In this study, correlations became weaker after adjustment for total energy intake. Previous studies have suggested that adjustment for energy intake increases the strength of correlations when variability of nutrient consumption is related to energy intake, but weakens the correlations when variability depends on systematic overestimation or underestimation [5]. In several other studies [7, 11, 17–19, 29–31], there was no improvement in the crude correlations for some nutrients after adjustment for energy intake, and systematic overestimation or underestimation is expected with the FFQ [31]. The high correlation of energy in this study was similar to the findings of another study, and the two methods used in both studies share some of the same sources of errors, such as the underestimation or overestimation (due to memory flaws) of the quantities of foods consumed, which could help explain the artificially high correlations of energy reported [7].

Regarding sex, the higher correlation in boys than in girls observed in this study was similar to that reported in a previous validation study among children [7]. The sex difference was possibly caused by gaps between the actual amount consumed and the portion size shown in the FFQ [32], which would be larger in girls than boys. Other factors considered would be that the under-reporting of diet among adolescent girls was associated with unstructured eating, concerns with self-image [33], and living in a city [34]. In addition, the correlation in children aged 7–9 years was higher than in those aged 10–17 years. This finding was different from the result of the reproducibility. We assumed that the portion size of FFQ might not be suitable for older children in Phnom Penh city. Furthermore, a larger discrepancy between the FFQ and 24Hs would be observed in cases where there were more opportunities to eat various types of foods among older children.

Cross-classification of the subjects was performed in order to achieve a measure of agreement between the two methods. With respect to total energy intake and the nutrients analyzed, 78% of the subjects were correctly classified by the FFQ into the same category as the 24Hs or the adjacent category, while a mean of 6% were grossly misclassified. The correct classification rate was similar to or higher than that obtained in a previous study [11] but lower than that of another study [20].

### Limitations and advantages

Several limitations of this study must be considered. First, both the FFQ and the 24H have similar sources of error, such as being reliant on subjects’ memory, and may be biased due to underestimation or overestimation [29]. Using the FFQ to assess the diet of school-aged children can also present greater methodological difficulties due to unfamiliarity of children with portion sizes, their limited knowledge of food names, their limited experience with food preparation, and their shorter attention span [14]. Second, this study was carried out by performing two surveys on the same day (i.e., the FFQ was completed after finishing the 24H), so the attention of the participants may have declined when they were responding to the FFQ. Third, vitamin D is an essential nutrient for optimal calcium absorption [2], but the FFQ did not assess this vitamin because we used a Nutrition Calculation Database, which lacked a method for handling vitamin D and some other nutrients. Thus, new software is needed to calculate the vitamin D intake.

Third, the student subjects were recruited from Phnom Penh city only. A further survey among students in other districts would be needed to validate the FFQ designed for all school-aged children of Cambodia.

A major strength of this study is that the FFQ was developed for school-aged children based on a survey that covered the whole of Cambodia and took into account foods commonly consumed by school-aged children. In addition, the portion size question was designed to be easy to answer, allowing children to point a finger at a particular portion size when asked to estimate consumption of a food.

## Conclusions

To our knowledge, this FFQ is the first one developed for Cambodia and the first study to provide evidence for the validity and reproducibility of an FFQ. The results of this study suggest that our new FFQ could be used to assess the dietary intake of school-aged children in Cambodia.

## Abbreviations

24H: 24-h recall survey
BMI: Body mass index
CAM-RDA: Recommended Dietary Allowances in Cambodia
DR: Dietary history record
FFQ: Food frequency questionnaire
FIDR: Foundation for International Development/Relief
FR: Food weight record
SD: Standard deviation
